# Residual Larvicidal Activity of Quinones against *Aedes aegypti*

**DOI:** 10.3390/molecules25173978

**Published:** 2020-08-31

**Authors:** Raquel L. Silva, Daniel P. Demarque, Renata G. Dusi, João Paulo B. Sousa, Lorena C. Albernaz, Laila S. Espindola

**Affiliations:** Laboratório de Farmacognosia, Universidade de Brasília, Campus Universitário Darcy Ribeiro, Brasília 70910-900, Brazil; rcl_unb@hotmail.com (R.L.S.); dpdemarque@gmail.com (D.P.D.); renatadusi@hotmail.com (R.G.D.); jpsousa595@gmail.com (J.P.B.S.); lorena.albernaz@gmail.com (L.C.A.)

**Keywords:** *Aedes aegypti*, residual larvicidal activity, *Connarus suberosus*, quinones, embelin, tectoquinone

## Abstract

The number of documented dengue cases has increased dramatically in recent years due to transmission through the *Aedes aegypti* mosquito bite. Vector control remains the most effective measure to protect against this and other arboviral diseases including Zika, chikungunya and (urban) yellow fever, with an established vaccine only available for yellow fever. Although the quinone class shows potential as leading compounds for larvicide development, limited information restricts the development of optimized structures and/or formulations. Thus, in this contribution we investigated the larvicidal and pupicidal activity of three quinone compounds isolated from a *Connarus suberosus* root wood ethyl acetate extract together with 28 quinones from other sources. Eight quinones demonstrated larvicidal activity, of which tectoquinone (**4**) proved to be the most active (LC_50_ 1.1 µg/mL). The essential residual effect parameter of four of these quinones was evaluated in laboratory trials, with tectoquinone (**4**) and 2-ethylanthraquinone (**7**) presenting the most prolonged activity. In small-scale field residual tests, tectoquinone (**4**) caused 100% larvae mortality over 5 days, supporting its selection for formulation trials to develop a prototype larvicide to control *Ae. aegypti*.

## 1. Introduction

Mosquitoes have the ability to transmit a variety of pathogenic agents responsible for causing diseases in humans and continue to be responsible for millions of deaths. Worldwide incidence of dengue has alarmingly risen 30 times in the last 30 years with an increasing number of countries reporting their first outbreaks. Zika, dengue, chikungunya and (urban) yellow fever viruses are all transmitted to humans through the *Aedes aegypti* mosquito bite [1]. The *Ae. aegypti* vector presents a worldwide threat once present in the majority of tropical and subtropical regions [2]. Despite great efforts, only the yellow fever vaccine is well-established. Furthermore, specific drug treatments are not available for dengue, chikungunya and Zika virus, so vector control remains the most effective preventative action against the aforementioned arboviral diseases [3,4].

According to the World Health Organization (WHO), vector control should be integrated, focusing on both the immature (egg, larva and pupa) and adult form of the mosquito. The WHO further classifies vector control into three broad strategies—biological, environmental and chemical [1]. For biological control, natural mosquito larval predators such as small fish and bacteria have been employed. Environmental or mechanical control consists of the elimination or active reduction of mosquito breeding sites through promoting awareness. Chemical control involves the use of insecticides against larvae and/or adult forms [1].

In recent years, the use of insecticides in crop pest control programs around the world has resulted in environmental damage, pest resurgence and resistance to insecticides, not to mention the lethal effects on non-target organisms [5,6]. Consequently, novel chemicals/compounds, including plant extracts, are being investigated. Plants provide a potential source of leading molecules [7,8]. In this context, natural product-based insecticides can be an alternative to the synthetic insecticides currently used, given numerous reports of mosquito resistance, non-target toxicity and environmental persistence. Furthermore, they can potentially be degraded more easily, less toxic to humans and other non-target organisms and less detrimental to the environment. Since larvicides are intended to be applied in water, plant-based product low bioavailability and persistence can indicate lower toxicity [9,10]. Although promising, research of natural-based insecticides is often limited to the laboratory scale, lacking reported studies of their application in a field setting [11].

*Connarus suberosus* Planch. (Connaraceae) demonstrates characteristics commonly associated with Cerrado biome plants, presenting a thick and corky bark, tortuous branches and dense pilosity [12]. The Cerrado is the second largest biome in Brazil, after the Amazon, comprising approximately 2 million km^2^, of which 44% is endemic flora [13]. Due to its rich biodiversity and high number of endemic species, the Cerrado biome is considered one of world’s hotspots for biodiversity preservation [14,15]. The Cerrado is home to more than 4800 plant and vertebrate species combined and contributes with almost half of Brazil’s surface water. Despite its environmental significance, 46% of its native vegetation has already been devastated, with deforestation rates 2.5 times higher than the Amazon biome between 2002 and 2011 [15].

In new larvicide development, it is essential to know the extent of the residual effects of the compound/formulation in order to determine the application frequency required to achieve effective vector control. The residual effect is the amount of time the sample remains active [16]. In the present study, we evaluate the larvicidal and pupicidal activity a total of 31 quinone compounds isolated from a *C. suberosus* extract and other sources together with the laboratory residual activity of 4 quinones for the first time. Furthermore, we conduct a small-scale field residuality trial and discuss the structural requirements of quinones in relation to larvicidal activity against *Ae. aegypti*.

## 2. Results

### 2.1. Chemical Profile and Compound Isolation

The *C. suberosus* ethyl acetate root wood extract active against third-instar *Ae. aegypti* larvae (100% mortality, 250 µg/mL) was pre-fractionated in a SPE DIOL column yielding five pre-fractions—A (hexane:dichloromethane, 9:1), B (dichloromethane:ethyl acetate, 20:1), C (100% ethyl acetate), D (ethyl acetate:methanol, 5:1) and E (100% methanol). Pre-fractions A and B were the most potent, both with 100% larvae mortality at 125 µg/mL.

The major peaks detected in high performance liquid chromatography (HPLC) of pre-fractions A, B, C and D presented similar chemical profiles (Figure S2). The para-benzoquinones—suberonone (**1**), rapanone (**2**) and embelin (**3**) were isolated from pre-fractions A, B and C (Figures S3–S14). In addition, the *C*-glycoside derivative of gallic acid—bergenin (**32**)—was isolated from pre-fractions C and D (Figures S15 and S16). Embelin (**3**) and rapanone (**2**) were active at 25 μg/mL against *Ae. aegypti* third-instar larvae, while suberonone (**1**) and bergenin (**32**) were inactive (Figure 1).

### 2.2. Larvicidal and Pupicidal Assays and Structural Aspects of Quinones

Larvicidal and pupicidal assays were performed following the WHO guideline recommendations. The samples were dissolved in dimethylsulfoxide (DMSO) and assays were performed in four replicates, repeated with a total of three different larvae batches. The number of dead larvae was recorded after 24 h and 48 h of exposure and the respective mortality percentage calculated.

The LC_50_ values were determined by diluting 5 to 6 concentrations of each compound (DMSO) in their activity range. Between 200 and 600 larvae were exposed in 4 replicates of 25 larvae. Larvae mortality was recorded after 24 h, 48 h and 72 h (Table 1). The temephos positive control and the vehicle negative control (DMSO) were carried out in parallel to ensure test validity.

**Figure 1:**
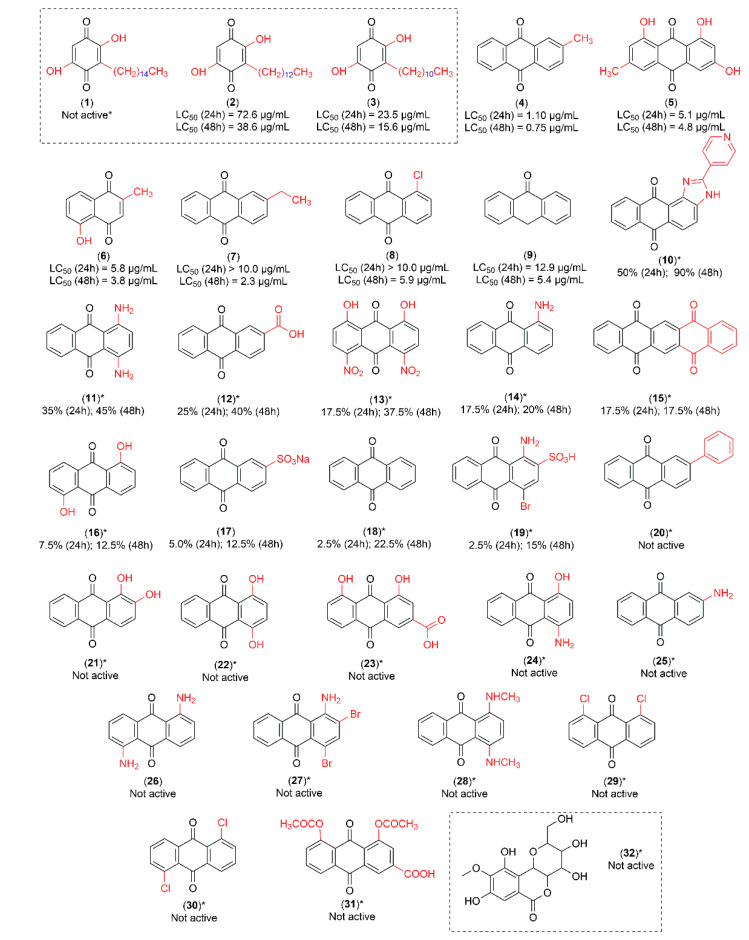
Quinone derivatives **1**–**31**: (**1**) suberonone; (**2**) rapanone; (**3**) embelin; (**4**) tectoquinone; (**5**) emodin; (**6**) plumbagin; (**7**) 2-ethylanthraquinone; (**8**) 1-chloroanthraquinone; (**9**) anthrone; (**10**) 2-(4-pyridil)-1*H*-anthra[1,2-*d*]imidazole-6,11-dione; (**11**) krisolamine; (**12**) anthraquinone-2-carboxylic acid; (**13**) 4,5-dinitrochrysazin; (**14**) 1-aminoanthraquinone; (**15**) 5,7,12,14-pentacenetetrone; (**16**) anthrarufin; (**17**) sodium anthraquinone-2-sulfonate; (**18**) anthraquinone; (**19**) bromaminic acid; (**20**) 2-phenylanthraquinone; (**21**) alizarin; (**22**) quinizarin; (**23**) rhein; (**24**) 1-amino-4-hydroxyanthraquinone; (**25**) 2-aminoanthraquinone; (**26**) 1,5-diaminoanthraquinone; (**27**) dibromoaminoanthraquinone; (**28**) 1,4-bis(methylamino)anthraquinone; (**29**) 1,8-dichloroanthraquinone; (**30**) 1,5-dichloroanthraquinone and (**31**) diacerein. *C*-glycoside derivative of gallic acid: (**32**) bergenin. All compounds tested against *Ae. aegypti* larvae at 25 µg/mL. LC_50_ values were only determined for compounds causing ≥80% mortality after 24 h (**2**–**9**). * Compounds with mortality percentages < 80% after 24 h.

All compounds were initially tested at 25 µg/mL, with LC_50_ values only determined for those with mortality percentages ≥ 80% after 24 h. For compounds with mortality percentages < 80% after 24 h, only the % mortality at 25 µg/mL was reported (Figure 1). 

In order to explore and understand the structural aspects of quinone larvicidal activity, we tested 28 quinone derivatives. The activities were measured after 24 h and 48 h (Figure 1). After preliminary larvicidal test results, the most active compounds were submitted to a dose-response curve to determine their respective LC_50_ and LC_90_ values (Table 1).

Regarding the isolated compounds from *C. suberosus*, embelin (**3**) presented an LC_50_ of 23.5 µg/mL, while rapanone (**2**) had an LC_50_ of 72.6 µg/mL (after 24 h). The most active commercial quinone was tectoquinone (**4**) with an LC_50_ of 1.1 µg/mL after 24 h.

### 2.3. Laboratory Residual Larvicide Activity

Laboratory residual activity was determined by replacing larvae in the containers every 24 h or 72 h until significant loss of activity. Based on previous results, residuality assays were performed by—A) an investigation of larvicidal activity with 24 h monitoring, with larvae replaced every 24 h until no longer effective; B) a 3-day cyclic study of larvicidal activity with 24 h assessments and larvae replaced every 72 h. The test was halted when 10–20% of the initial mortality effect was observed as this was considered a significant reduction in residual larvicidal activity. Positive (temephos 0.025 µg/mL) and negative (<1.0% DMSO in tap water) controls were performed in parallel.

Although *C. suberosus* was the initial main source of compounds in the present study, the isolated natural compounds had lower larvicidal activities than the commercial quinone derivatives. Therefore, from the eight most active compounds, we selected—tectoquinone (**4**); 2-ethylanthraquinone (**7**); 1-chloroanthraquinone (**8**) and anthrone (**9**) for residuality tests based on larvicidal activity and quantity available (Figures S17–S24).

**Figure 2:**
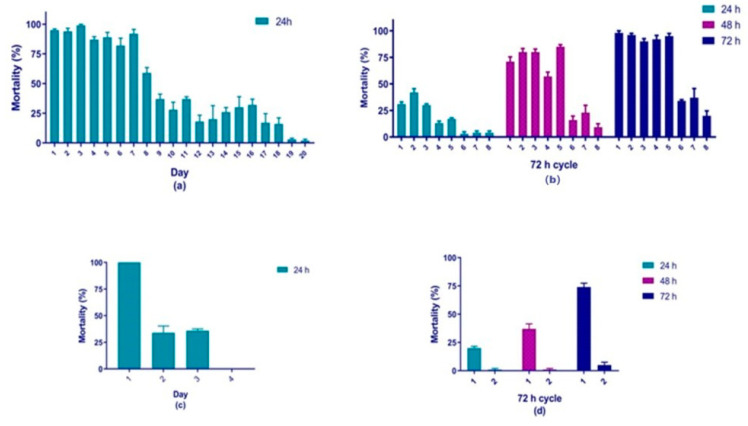
(**a**) Tectoquinone (**4**) laboratory residual larvicide effect after 20 days. Larvae mortality was recorded after 24 h, with larvae replaced every 24 h. (**b**) 2-ethylanthraquinone (**7**) laboratory residual larvicide effect after 8 cycles of 72 h. Larvae mortality was recorded after 24 h, with larvae replaced every 72 h. (**c**) Anthrone (**9**) laboratory residual larvicide effect after 4 days. Larvae mortality was recorded after 24 h, with larvae replaced every 24 h. (**d**) 1-chloroanthraquinone (**8**) laboratory residual larvicide effect after 3 cycles of 72 h. Larvae mortality was recorded after 24 h, with larvae replaced every 72 h. Error bars = standard error of the mortality mean value. DMSO (negative control) mortality <5%. Temephos (positive control) mortality decreased from 96% (Day 1) to 68% (Day 4) and to <48% (Day 9).

Tectoquinone (**4**) and 2-ethylanthraquinone (**7**) showed the longest residual effects. Regarding the LC_50_ values, the highest mortality was observed for tectoquinone (**4**) (3.5 µg/mL) which presented 95% mortality on Day 1 (after 24 h) which decreased to 37% on Day 9 (Figure 2a). 2-ethylanthraquinone (**7**) (10 µg/mL), displayed 98% mortality after the first 72 h cycle (Day 3), which reduced to 34% at the end of the sixth 72 h cycle (Day 18) (Figure 2b). The mortality induced by **9** decreased from 100% (Day 1) to 30% after 48 h (Day 2) (Figure 2c). Compound **8** presented 74% mortality after the first 72 h cycle (Day 3) which reduced to 5% after Day 6 (Figure 2d). The mortality induced by temephos decreased from 96% (Day 1) to 68% (Day 4) and to <48% (Day 9).

Regarding residuality results, it is the first time that residual activity against third-instar *Ae. aegypti* larvae has been reported for tectoquinone (**4**), 2-ethylanthraquinone (**7**), 1-chloroanthraquinone (**8**) and anthrone (**9**). Compounds **4** and **7** showed the most promising results as their mortality percentages remained at >80% until Day 7 (Figure 2a) and >90% until Day 15 (Figure 2b), respectively.

### 2.4. Small-Scale Field Residual Larvicide Activity

Small-scale field residual larvicide activity was performed in plastic containers filled with 10 L of tap water. Positive (temephos 0.013 µg/mL) and negative (<1.0% DMSO in tap water) controls were performed in parallel.

The entire larval batch (live and dead) was replaced every 24 h until mortality was reduced by less than 80%, an initial laboratory screening limit.

**Figure 3:**
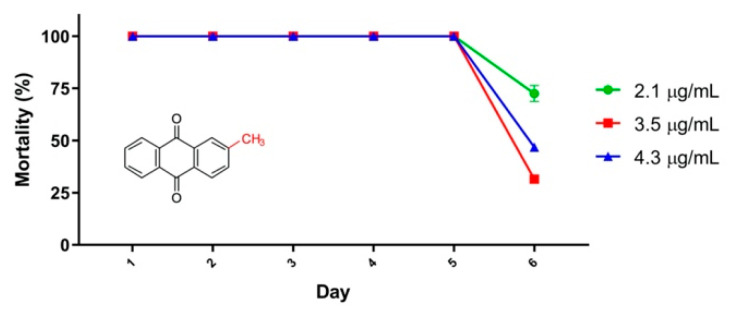
Tectoquinone (**4**; 2.1 µg/mL, 3.5 µg/mL and 4.3 µg/mL) small-scale field residual larvicide effect over 6 days. Larvae mortality was recorded every 24 h, with daily larvae replacement. DMSO (negative control) mortality < 5%. Temephos (positive control) mortality decreased from 99% (Day 1) to 85% (Day 6) and to <56% (Day 9). Error bars = standard error of the mortality mean value.

A small-scale field test monitoring residual larvicide activity was conducted with tectoquinone (**4**) as it demonstrated the highest mortality from 24 h until Day 7 in the laboratory test (Figure 2a). Tectoquinone showed 100% mortality for the 3 concentrations tested (2.1 µg/mL, 3.5 µg/mL and 4.3 µg/mL) until Day 5 in the small-scale field test. Larvae mortality decreased >20% on Day 6 (laboratory limit) and the test was halted. The LC_90_ concentration (2.1 µg/mL) demonstrated the most activity (72.5% mortality) on Day 6, while the higher concentrations caused lower mortality on the same Day (Figure 3) which can be explained by precipitation observed during the test. The lowest concentration was therefore considered for field trial formulation development. The mortality induced by temephos decreased from 99% (Day 1) to 85% (Day 6) and to <56% (Day 9).

Larvae mortality caused by tectoquinone (**4**) was higher in the small-scale field tests (100%, see Figure 3) than in the laboratory test (87% to 99%, see Figure 2a) for the first 5 days. This may be due to the environmental conditions.

### 2.5. Pupicidal Test

None of the compounds displayed pupicidal activity after 24 h exposure, suggesting a mechanism of action specific to larvae or lack of bioavailability for pupae.

## 3. Discussion

A study with a *C. suberosus* root bark conducted by our research group obtained a mixture of rapanone and previously unreported suberonone [17]. The present study constitutes the first investigation of larvicidal activity of separated suberonone (**1**) and rapanone (**2**), together with the first isolation of embelin (**3**) and bergenin (**32**) from *C. suberosus* root wood, following the assay criteria specified in the WHO guideline [18]. It is not common to find related scientific publications that followed the WHO criteria. This standardized methodology affords more reliability in terms of structure-activity correlation which can be used in future studies for lead compound optimization. A potassium salt of embelin mixed with rapanone, isolated from *Rapanea melanophloeos* (Myrsinaceae), was tested against *Ae. aegypti* larvae with an LC_50_ of 2.4 µg/mL after 48 h exposure [19]. The study concluded that some para-benzoquinones present in plants, such as embelin (mixed with rapanone), myrsinone and myrsinaquinone, demonstrated 90% lethality against second-instar *Ae. aegypti* larvae at 4–5 µg/mL. However, the methodology employed was not in accordance with the WHO guideline [18] and a lower number of larvae were tested. Sousa and co-authors reported 6 larvicidal para-benzoquinones in which the unsubstituted para-benzoquinone (without hydroxyl or alkyl groups) exhibited the lowest LC_50_ (90 µg/mL), while 2-isopropyl-para-benzoquinone (alkyl group 3C-isopropyl) was more active after 24 h (LC_50_ 33 µg/mL). In general, the presence of alkyl groups is associated with increased larvicidal activity which is modulated by their number, position and size [20]. In the present study, embelin (**3**), which possesses an alkyl chain with 11-C, presented higher larvae mortality (LC_50_ 23.5 µg/mL) than the 2-isopropyl-para-benzoquinone [19]. However, mortality decreased as the alkyl chain lengthened—rapanone (**2**; alkyl group 13C) and suberonone (**1**; alkyl group 15C). This suggests that the impact of alkyl chain length is limited.

We observed that the methyl group directly attached to the aromatic ring of the anthracene nucleus is relevant and has an important role in larvicidal activity. The most active anthraquinone—tectoquinone (**4**; LC_50_ 1.1 µg/mL after 24 h)—has a methyl group substituent at the C-2 position. A decrease in larvicidal activity was observed in comparison to 2-ethylanthraquinone (**7**; LC_50_ > 10 µg/mL after 24 h), which has an ethyl group at the same C-2 position. Moreover, anthraquinone (**18**), without any substituent, did not exhibit larvicidal activity after 24 h. Emodin (**5**; LC_50_ 5.0 µg/mL after 24 h), which also possesses a methyl group directly attached to the aromatic ring of the anthracene nucleus at the C-6 position, presented the second highest larvicidal activity of the quinones.

Cheng et al. (2008) reported activity against fourth-instar *Ae. aegypti* larvae for—tectoquinone (LC_50_ 3.3 µg/mL and LC_90_ 8.8 µg/mL)*;* 9,10-anthraquinone (LC_50_ and LC_90_ > 25 µg/mL); emodin (LC_50_ 5.3 µg/mL and LC_90_ 19.1 µg/mL); alizarin (LC_50_ and LC_90_ > 25 µg/mL) and anthraquinone-2-carboxylic acid (LC_50_ 16.3 µg/mL and LC_90_ 25 µg/mL). The authors concluded that comparisons of larvicidal activity of anthraquinone congener skeletons demonstrated stronger activity when there was a methyl group at the C-2 position, as in tectoquinone [21].

In the present tectoquinone (**4**) data, we determined—LC_50_ 1.1 µg/mL and LC_90_ 2.1 µg/mL. It is important to note that the larvicidal assays performed in this study differed considerably from the aforementioned 2008 study [21]—number of quinones tested (31 vs. 7), larvae stage tested (L3 vs. L4), total number of larvae tested (1800 vs. 360), final test volume (120 mL vs. 25 mL), number of larvae in each recipient (25 vs. 10), number of replicates (4 using 3 larvae batches vs. 4), duration (24, 48 and 72 h vs. 24 h) and protocol (WHO, 2005 vs. Momin & Nair, 2001 [22]). In addition, not only did our investigation of 31 quinones follow the WHO criteria, it also constituted the first report of tectoquinone, 2-ethylanthraquinone, anthrone and 1-chloroanthraquinone residual activity against third-instar *Ae. aegypti* larvae.

As supported by previous studies, emodin shows a variety of biological activities, including laxative properties, similar to rhein. Both compounds present a hydroxyl group at C-1 and C-8 positions but differ in that emodin possesses a methyl group (C-6), whereas rhein contains a carboxyl group (C-3) [23]. However, in the present study, it was possible to verify that the larvicidal activity of emodin (**5**; LC_50_ 5.0 µg/mL and LC_90_ 10 µg/mL after 24 h) and rhein (**23**; inactive at 25 µg/mL) were not similar. These results suggest that larvicidal activity cannot be linked to the presence of hydroxyl groups. This conclusion is consistent with Yang et al. (2003) [24] and Cheng et al. (2008) [21].

It is noteworthy that hydroxyl groups play a key role in commercially available laxatives containing hydroxyanthracene derivatives [25]. Several studies indicate that these hydroxyanthracenic derivatives present toxic side effects associated with the hydroxyl group position (C-1/C-4 and C-8/C-5) in the anthracene nucleus [26,27,28]. The absence of hydroxyl groups in larvicidal anthraquinones, with the exception of emodin, suggests lower toxicity for humans.

There are numerous mechanisms of action described for quinones in the literature [29]. More specifically, in insects, juglone—a quinone derivative—showed competitive inhibition with both glutathione *S*-transferase and 1-chloro-2,4-dinitrobenzene [30], while emodin exerts antifeedant activity and post-ingestive damage in the midgut [31]. Finally, mitochondrial complex III inhibition by quinones was described [32]. However, more studies are needed to better understand the mechanism of action of quinones in *Ae. aegypti*.

According to the European Chemicals Agency (ECHA) registration information, tectoquinone (**4**) is not hydrolysable in water and as such is not expected to be readily biodegradable. However, this chemical does not bioaccumulate in the food chain or persist in the soil environment. The exposure risk to soil-dwelling animals is considered moderate to low. The 37.5-day half-life (estimated by the Estimation Programs Interface, 2018) indicates that the chemical is not persistent in water and the exposure risk to aquatic animals is also considered moderate to low [33].

The small-scale field residual larvicide activity results showed that larvae mortality remained at 100% until Day 5. This study analyzed the compound, only without any kind of formulation. It may, however, be possible to increase the number of days using a controlled-release formulation. In addition, the short half-life could also be addressed in a similar manner once it is possible to achieve a steady state situation.

Residual effect data is fundamental for authorities to consider the logistics of mosquito control. The Brazilian Ministry of Health is responsible for the acquisition of insecticides used in control programs targeting mosquito disease vectors. The current guideline specifies larvicide application at 2-monthly intervals, only in households or public places in which there is no other means of eliminating the breeding site [34]. Therefore, formulation studies aim to develop a safe prototype with a prolonged residual effect.

## 4. Materials and Methods

### 4.1. Chemicals and Instrumental Analysis

The organophosphate insecticide temephos was purchased from Sigma Aldrich (Buchs, Switzerland). Quinones were purchased from Alfa Aesar (Ward Hill, MA, USA). All other chemicals used in this study were of high performance liquid chromatography (HPLC) grade and are commercially available.

^1^H NMR (nuclear magnetic resonance of hydrogen), HSQC (heteronuclear single quantum correlation) and HMBC (heteronuclear multiple bond correlation) spectra were recorded on a 600 MHz nuclear magnetic resonance (NMR) spectrometer (Bruker, Rheinstteten, Germany), while ^13^C spectra were recorded on a 300 MHz spectrometer (Varian, Palo Alto, CA, USA), with tetramethylsilane used as an internal standard. Molecular weight was determined using HRMS (high resolution mass spectrometry) (qTOF-quadrupole-time-of-flight) (Bruker, Bremen, Germany).

### 4.2. Plant Material

*Connarus suberosus* Planch. (Connaraceae) was collected in 2010 from the Cerrado biome in the Lagoa Formosa area, Planaltina, Distrito Federal, Brazil, south latitude 15°27′34.2′′; south longitude 47°92′3.3′′; at an altitude of 1071 m. Identification was subsequently performed by a botanist—Prof. José Elias de Paula. A voucher specimen was deposited in the Universidade de Brasília (UnB) Herbarium, under the reference J. Elias de Paula (UB) 3820. Root wood was separated, dried, pulverized and extracted by maceration with ethyl acetate. The extractive solution was concentrated in a rotary evaporator, yielding the crude extract, which was stored at −20 °C.

### 4.3. Extraction and Isolation

The *C. suberosus* ethyl acetate root wood extract active against third-instar *Ae. aegypti* larvae (100% mortality, 250 µg/mL, 3.6 g) was pre-fractionated in a DIO Spe-ed SPE cartridges (Applied Separations, Allentown, PA, USA) in five pre-fractions—A (hexane and dichloromethane, 9:1), B (dichloromethane and ethyl acetate, 20:1), C (100% ethyl acetate), D (ethyl acetate and methanol, 5:1) and E (100% methanol). Pre-fractions A (349.1 mg) and B (645.8 mg) were the most potent, both with 100% larvae mortality at 125 µg/mL. Pre-fraction B displayed the cleanest HPLC profile and presented the highest yield. Pre-fraction B was thus fractionated using a semi-preparative 1525 HPLC (Waters, Milford, MA, USA). The column was SunFire C18 (Waters, Dublin, Ireland), 5 µm particle size, 10 × 250 mm. The mobile phase was water and methanol: 65:35 (0–4 min); 0:100 (4–21 min) and 65:35 (21–30 min) by volume, at a flow rate of 4 mL/min. Three fractions were collected and identified as—(**1**) suberonone [17] (2,5-dihydroxy-3-entadecylcyclohexa-2,5-diene-1,4-dione) (RT = 24.2 min, yield 0.46%); (**2**) rapanone [17] (2,5-dihydroxy-3-tridecylcyclohexa-2,5-diene-1,4-dione) (RT = 22.0 min, yield 3.3%) and (**3**) embelin [35] (2,5-dihydroxy-3-undecylcyclohexa-2,5-diene-1,4-dione) (RT = 20.1 min, yield 5.6%). Pre-fractions A and C were studied using the same procedures and yielded the same compounds—**1**, **2** and **3**, while bergenin (**32**) [36] (*C*-glycoside of 4-*O*-methyl gallic acid) was isolated from pre-fractions C and D (Figure S1).

### 4.4. Biological Assays

Third-instar *Ae. aegypti* larvae (Rockefeller strain) aged 72–96 h and pupae older than 168 h were collected from a mosquito colony maintained at the Laboratório de Farmacognosia Insectarium at the Universidade de Brasília without exposure to any insecticide to perform all biological assays. The mosquitoes were maintained at 28 ± 2 °C and 70 ± 10% relative humidity (RH) and a photoperiod of 12 h. Egg hatching occurred in shallow tap water and fish food was added. Adult insects were fed with 10% sugar solution-soaked filter paper (Whatman, Canterbury, UK), changed twice a week. A mare blood meal (Hospital Veterinário of the Universidade de Brasília) was given three times a week to allow egg production.

Larvicidal assays were performed following the WHO guideline recommendations [18] with modifications. Initially, the crude extract (250 μg/mL) was dissolved in dimethylsulfoxide (DMSO) and placed in 12-well plates. A total of 120 larvae were exposed in 4 replicates of 10 larvae, for each well with a 3 mL final volume, three times. After 24 h and 48 h of exposure, the number of dead larvae was recorded and the mortality percentage calculated. Larvae with no movement, confirmed by light plate agitation, were considered dead. Positive (temephos) and negative (<1.0% DMSO in tap water) controls were performed in parallel Pre-fractions (125 μg/mL), fractions (100 μg/mL), natural (25 μg/mL) and commercial (25 μg/mL) compounds were tested following the same procedure, with DMSO as a negative control.

Based on the percentage mortality (≥80%) of natural and commercial compounds, the LC_50_ (µg/mL) values and their respective 95% fiducial limits, together with their lower/upper confidence interval, were determined. The temephos positive control (0.0016 to 0.0250 µg/mL in tap water) was carried out in parallel to ensure test validity. For each bioassay, 25 third-instar larvae were transferred to cups containing a final volume of 20 mL or 120 mL of tap water together with the sample tested at the desired concentration.

The LC_50_ values were calculated by diluting 5 to 6 concentrations of each compound (DMSO) in their activity range. A total of 200 to 600 larvae were exposed in 4 replicates of 25 larvae, at each concentration, using three larvae lots from the same colony. Larvae mortality was recorded after an exposure of 24 h, 48 h or 72 h (Table 1). For each bioassay, the temperature was maintained at 28 ± 2 °C and 70 ± 10% RH, with a 12 h photoperiod.

Pupicidal screening assays were performed with crude extracts (250 μg/mL), natural (50 μg/mL) and commercial (25 μg/mL) compounds dissolved in DMSO and placed in a covered cup containing a final volume of 20 mL of tap water. Four cups were tested per concentration (10 pupae per cup) with pupal mortality recorded after 24 h exposure. A total of 40 pupae were exposed in 4 replicates of 10 pupae per sample.

Laboratory residual activity was determined by comparing the number of live and dead larvae in treated and control assays. For each bioassay, 25 third-instar larvae were transferred to each of 4 cups containing a final volume of 120 mL of tap water, together with the sample tested at the desired concentration (the lowest concentration observed to cause the highest mortality in previous larvicidal activity tests). Each compound was diluted in DMSO which was also used as the negative control (maximum 1.0% in tap water). Positive (temephos 0.025 µg/mL) control was performed in parallel.

During the high number of assays conducted to determine the LC_50_ and LC_90_ values, we observed different activity—some compounds achieved maximum mortality after 24 h while others after 72 h. Therefore, we performed the residuality assays in two different ways—(1) larvae mortality was recorded after 24 h, with larvae replaced every 24 h until no longer effective and (2) a 3-day cyclic study of larvicidal activity with 24 h assessments and larvae replaced every 72 h.

The test was halted when 10–20% of the initial mortality effect was observed as this was considered a significant reduction in residual larvicidal activity in accordance with the WHO guideline recommendations [18].

Small-scale field residual larvicide activity was performed in plastic containers filled with 10 L of tap water. After 24 h, 30 third-instar *Ae. aegypti* larvae were added to each container. After 2 h of acclimation, tectoquinone (**4**) diluted in DMSO was added to each container to achieve three different exposure concentrations—2.1 µg/mL (LC_90_ value), 3.5 µg/mL (LC_90_ value × 1.5) and 4.3 µg/mL (LC_90_ value × 2). DMSO was used as the negative control (maximum 1.0% in tap water). Each concentration and negative control were tested in quadruplicate. A positive (temephos 0.013 µg/mL) control was performed in parallel. Containers were covered with a nylon mesh screen. Each container was examined after 24 h and the number of live larvae counted to determine post-treatment larval mortality. The entire larval batch (live and dead) was replaced every 24 h until mortality was reduced by less than 80%, an initial laboratory screening limit.

### 4.5. Statistical Analysis

The average larvae mortality data were subjected to GraphPad Prism 8 analysis to calculate the LC_50_, LC_90_ and other statistics at 95% fiducial limits of lower and upper confidence. In order to determine the intrinsic activity of each larvicide, 3 replicates using larvae from different rearing batches were made at different times with the results pooled for analysis (*n* = 300 larvae per dose). According to the WHO guidelines, tests with a control mortality > 5% must be corrected using Abbott’s formula and tests with control mortality > 20% must be discarded.

## 5. Conclusions

This study is the first report of the isolation and larvicidal activity of suberonone (**1**), rapanone (**2**), embelin (**3**) and bergenin (**32**) from *Connarus suberosus* root wood.

A total of 31 quinone derivatives were investigated for activity against *Ae. aegypti* larvae and pupae, with 8 compounds demonstrating larvicidal activity of >80% at 25 µg/mL. Of the three quinones isolated from *C. suberosus*, embelin (**3**) proved the most active, while tectoquinone (**4**) was the most potent of the remaining 28 quinones from other sources. Furthermore, this is the first report of residual activity against third-instar *Ae. aegypti* larvae for tectoquinone (**4**), 2-ethylanthraquinone (**7**), 1-chloroanthraquinone (**8**) and anthrone (**9**), with **4** and **7** showing the most promising results.

The properties of tectoquinone (**4**) underline the suitability of this chemical as a candidate to develop a larvicidal prototype—100% larvae mortality over 5 days, non-hydrolysable, no bioaccumulation in the food chain or persistence in the soil environment. Formulation and non-target organism studies are currently being performed to develop a non-toxic prototype larvicide with prolonged activity to control *Ae. aegypti*.

## Figures and Tables

**Table 1:** LC_50_ (µg/mL) and LC_90_ (µg/mL) data for compounds **2**–**9**.

Compound	Concentration (µg/mL)	^a^ FV (mL)	^b^ n	^c^ N	LC_50_ µg/mL 24 h *, 48 h **, 72 h *** (95% ^d^ CI)	LC_90_ µg/mL 24 h *, 48 h **, 72 h *** (95% CI)
**2**	200; 100; 50; 25; 12.5	3	600	12	72.62 * (65.27–81.02) 38.63 ** (35.47–42.14)	>200.0 * 77.95 ** (65.59–92.02)
**3**	50; 25; 20; 15; 10; 5	20	1800	12	23.51 * (22.12–25.16) 15.55 ** (14.64–16.51)	48.36 * (40.66–>50.0) 26.51 ** (23.39–30.05)
**4**	3.5; 1.75; 0.88; 0.44; 0.22; 0.11	120	1800	12	1.10 * (1.04–1.17) 0.75 ** (0.70–0.82) 0.53 *** (0.49–0.57)	2.11 * (1.86–2.39) 1.71 ** (1.45–2.01) 1.31 *** (1.10–1.56)
**5**	50; 25; 12.5; 6.25; 3.13; 1.56	20	1800	12	4.99 * (4.52–5.50) 4.70 ** (4.35–5.09)	10.05 * (8.31–12.13) 7.57 ** (6.78–8.42)
**6**	25; 12.5; 6.25; 3.13; 1.56; 0.78	20	1800	12	6.52 * (6.17–6.88) 4.91 ** (4.45–5.41)	12.70 * (11.18–14.39) 10.84 ** (8.88–13.13)
**7**	10; 4; 1.6; 0.64; 0.26; 0.10	120	1800	12	>10.0 * 2.27 ** (1.99–2.59) 1.28 *** (1.19–1.37)	>10.0 * 8.30** (6.24–>10.0) 3.06*** (2.66–3.50)
**8**	10; 1; 0.1; 0.01	120	1200	12	>10.0 * 5.98 ** (5.31–6.76) 2.80 *** (2.18–3.58)	>10.0 * >10.0 ** >10.0 ***
**9**	150; 37.5; 9.38; 2.34; 0.59; 0.5	120	1800	12	12.87 * (10.13–16.38) 5.44 ** (4.16–7.15) 1.83 *** (1.48–2.27)	>150.0 * 107.4 ** (58.83–>150.0) 17.09 *** (10.79–26.75)
**temephos**	0.025; 0.0125; 0.00625; 0.003125; 0.0015625	120	1500	12	0.0081 * (0.0076–0.0085) 0.0064 ** (0.0063–0.0068) 0.0054 *** (0.0055–0.0060)	0.0130 * (0.0117–0.0144) 0.0107 ** (0.0096–0.0121) 0.0096 *** (0.0088–0.0109)

^a^ FV: final volume. ^b^ n: number of larvae. ^c^ N: number of replicates. ^d^ CI: lower/upper confidence interval. DMSO (negative control)—larvae mortality < 20%. According to the WHO guideline, tests with control mortality > 20% were discarded.

## Supplementary Materials

The following are available online, Figure S1: Extraction flow of suberonone (2), rapanone (2), embelin (3) and bergenin (32); Figure S2: Analytical chromatograms of the pre-fractions A, B, C and D. Peaks related to bergenin (32), embelin (3) e rapanone (2). Suberonone (1) was only observed with a semi-prepared column; Figure S3: Suberonone (1) RMN 1H (600 Mz) in DMSO-d6; Figure S4: Suberonone (1) RMN 13C (600 Mz) in DMSO-d6; Figure S5: Suberonone (1) MS; Figure S6: Suberonone (1) IR; Figure S7: Rapanone (2) RMN 1H (600 Mz) in DMSO-d6; Figure S8: Rapanone (2) RMN 13C (300 Mz) in DMSO-d6; Figure S9: Rapanone (2) MS; Figure S10: Rapanone (2) IR; Figure S11: Embelin (3) RMN 1H (600 Mz) in DMSO-d6; Figure S12: Embelin (3) RMN 13C (600 Mz) in DMSO-d6; Figure S13: Embelin (3) MS; Figure S14: Embelin (3) IR; Figure S15: Bergenin (32) RMN 1H (600 Mz) in MeOD; Figure S16: Bergenin (32) RMN 13C (300 Mz) in MeOD; Figure S17: Rapanone (2) LC50 (µg/mL). Final Volume = 3 mL. Number of larvae = 600; Figure S18: Embelin (3) LC50 (µg/mL). Final Volume = 20 mL. Number of larvae = 1800; Figure S19: Tectoquinone (4) LC50 (µg/mL). Final Volume = 120 mL. Number of larvae = 1800; Figure S20: Emodin (5) LC50 (µg/mL). Final Volume = 20 mL. Number of larvae = 1800; Figure S21: Plumbagin (6) LC50 (µg/mL). Final Volume = 20 mL. Number of larvae = 1800; Figure S22: 2-ethylanthraquinone (7) LC50 (µg/mL). Final Volume = 120 mL. Number of larvae = 1800; Figure S23: 1-chloroanthraquinone (8) LC50 (µg/mL). Final Volume = 120 mL. Number of larvae = 1200; Figure S24: Anthrone (9) LC50 (µg/mL). Final Volume = 120 mL. Number of larvae = 1800; Figure S25: Temephos (positive control) LC50 (µg/mL). Final Volume = 120 mL. Number of larvae = 1500.
