# Supplementary material for: Residual Larvicidal Activity of Quinones against Aedes aegypti
Source: Molecules. 2020 Aug 31;25(17):3978. doi: 10.3390/molecules25173978 (PMC7504811; doi:10.3390/molecules25173978)
Supplement: Supplementary file 1 [file molecules-25-03978-s001.pdf]

Supplementary Materials

# Residual larvicidal activity of quinones against *Aedes aegypti*

**Raquel L. Silva [1], Daniel P. Demarque [1], Renata G. Dusi [1], João Paulo B. Sousa [1], Lorena C. Albernaz [1] and Laila S. Espindola [1,*]**

[1] Laboratório de Farmacognosia, Universidade de Brasília, Campus Universitário Darcy Ribeiro, Brasília 70910-900, Brazil; rcl_unb@hotmail.com (R.L.S.); dpdemarque@gmail.com (D.P.D.); renatadusi@hotmail.com (R.G.D.); jpsousa595@gmail.com (J.P.B.S.); lorena.albernaz@gmail.com (L.C.A.)

* Correspondence: darvenne@unb.br (L.S.E.)

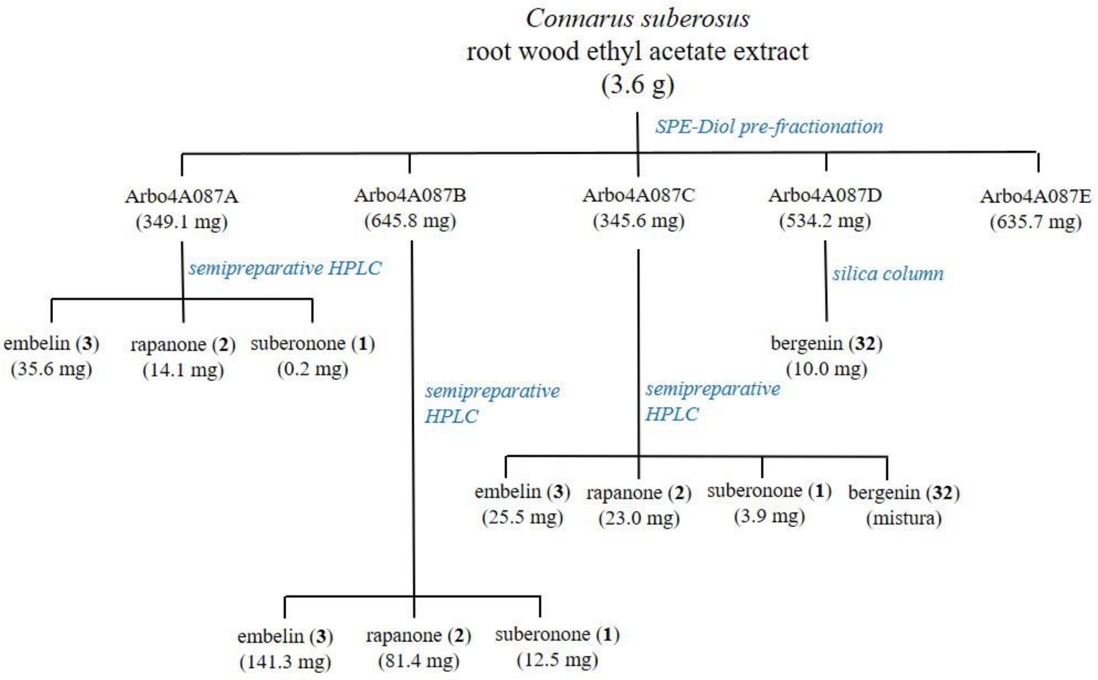


**Figure S1**. Extraction flow of suberonone (**1**), rapanone (**2**), embelin (**3**) and bergenin (**32**).

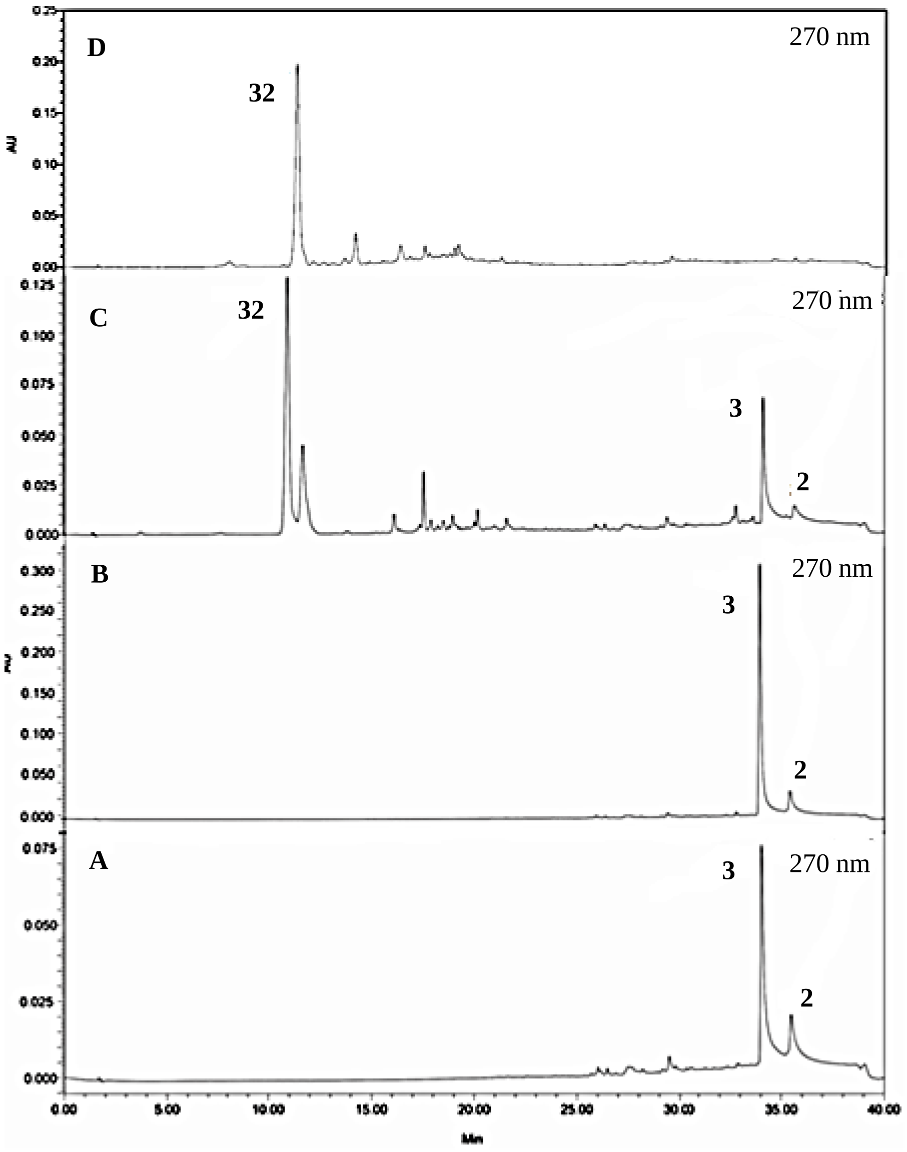


**Figure S2.** Analytical chromatograms of the pre-fractions A, B, C and D. Peaks related to bergenin (**32**), embelin (**3**) and rapanone (**2**). Suberonone (**1**) was only observed with a semi-prepared column.

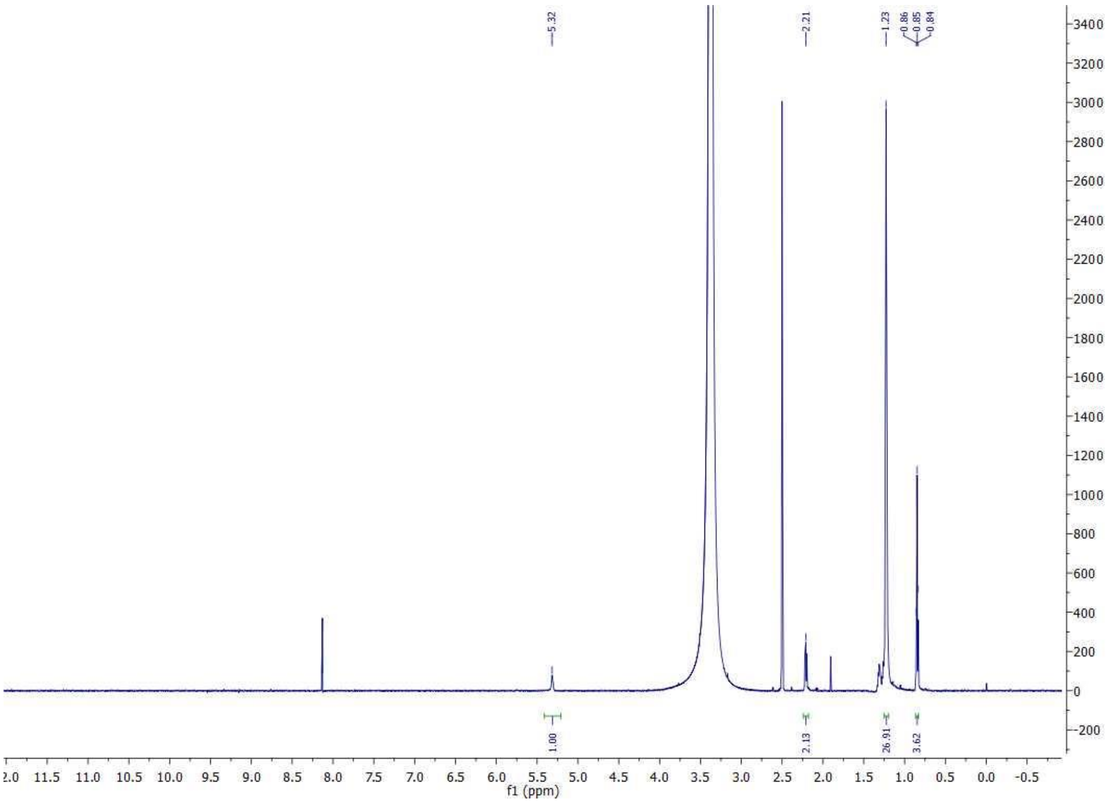


**Figure S3**. Suberonone (**1**) RMN 1H (600 Mz) in DMSO-d6.

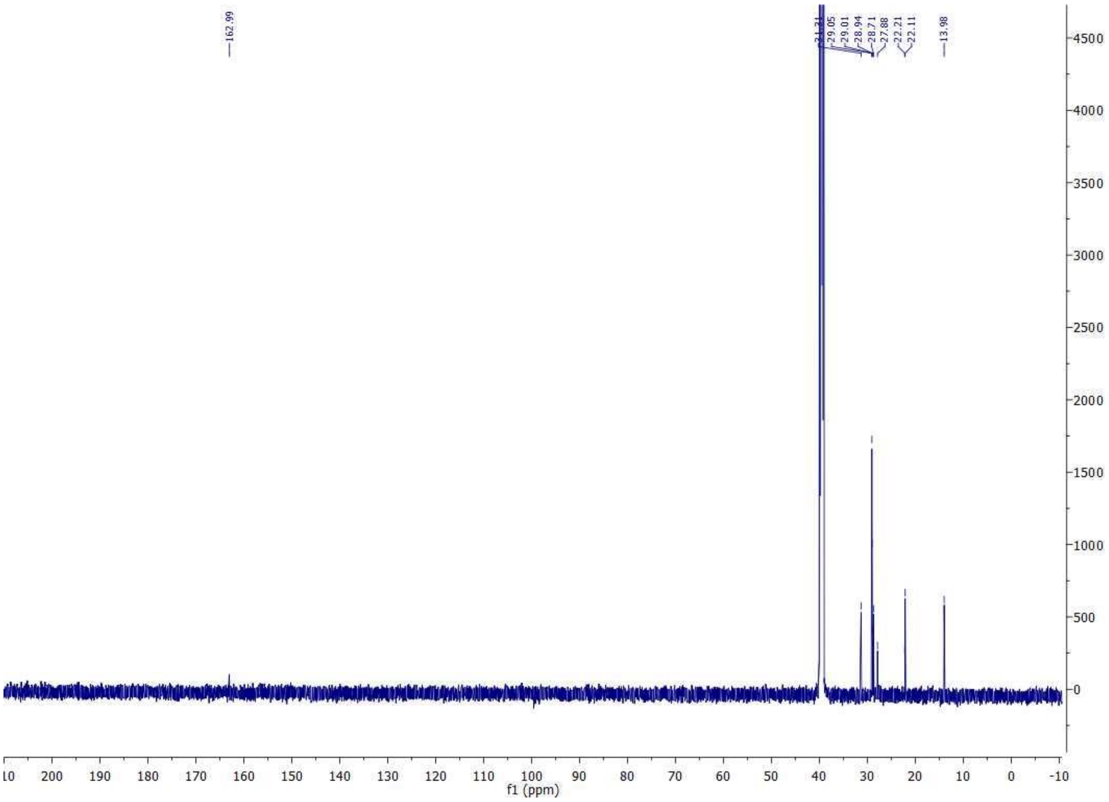


**Figure S4.** Suberonone (**1**) RMN 13C (600 Mz) in DMSO-d6.

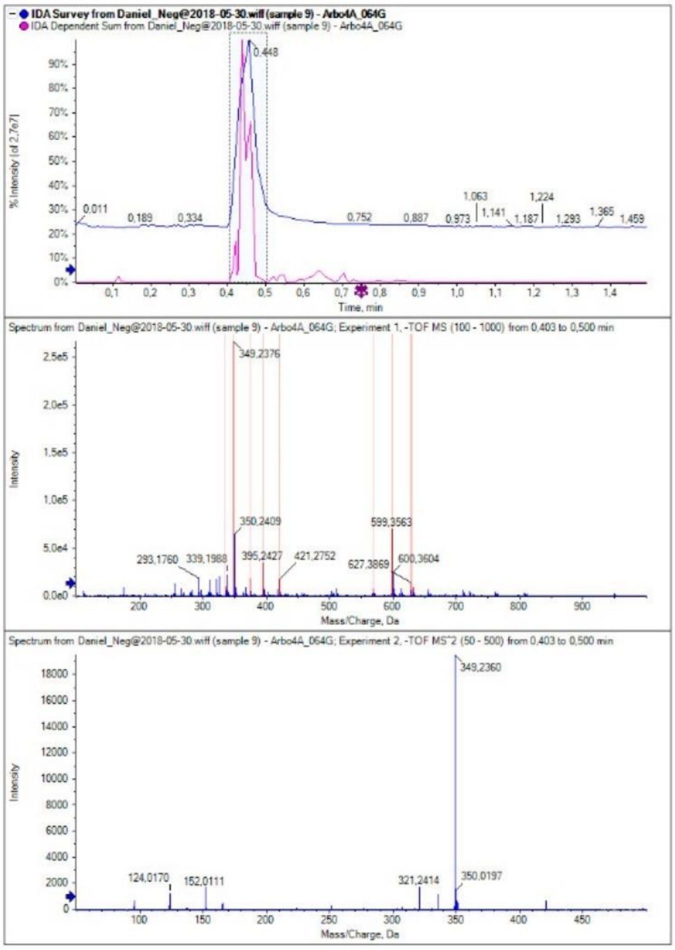


05/06/2018 17:51:52

**Figure S5.** Suberonone (**1**) MS.

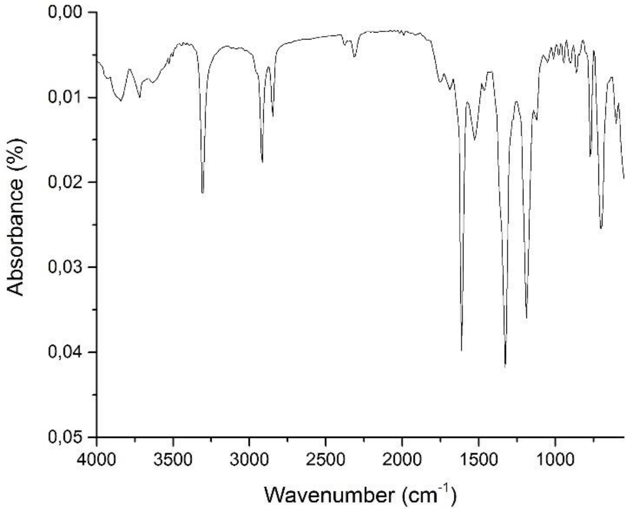


**Figure S6.** Suberonone (**1**) IR.

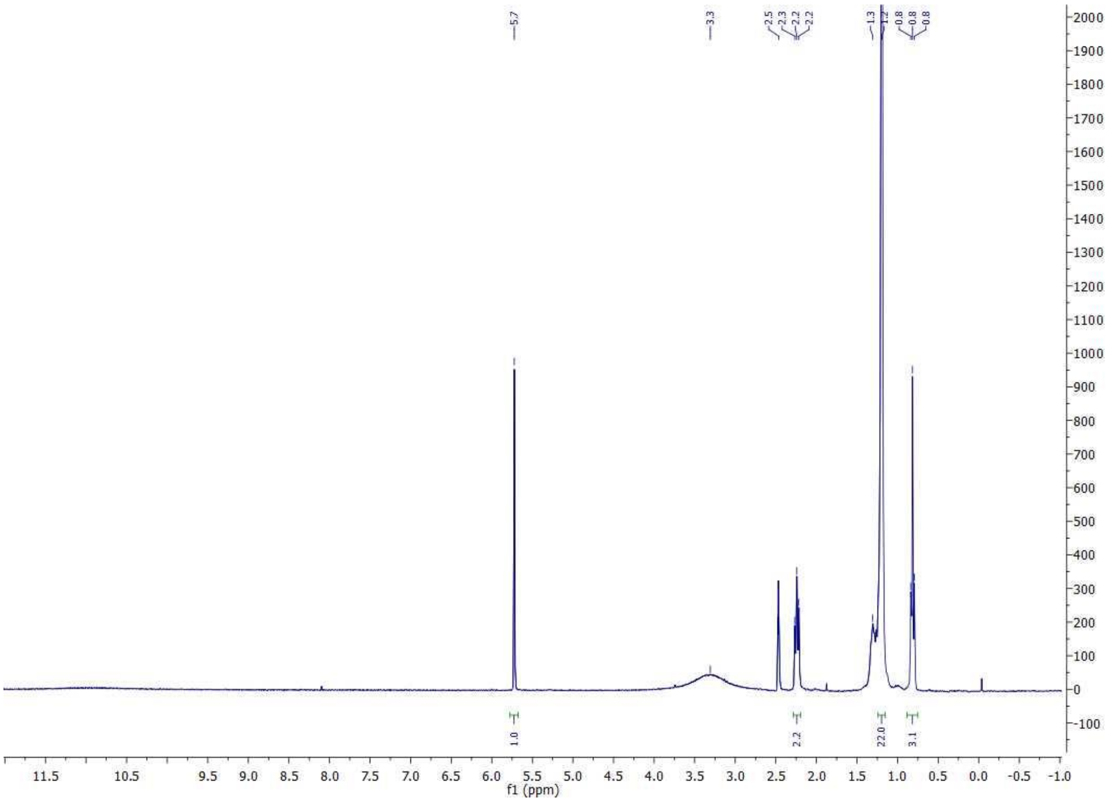


**Figure S7**. Rapanone (**2**) RMN 1H (600 Mz) in DMSO-d6.

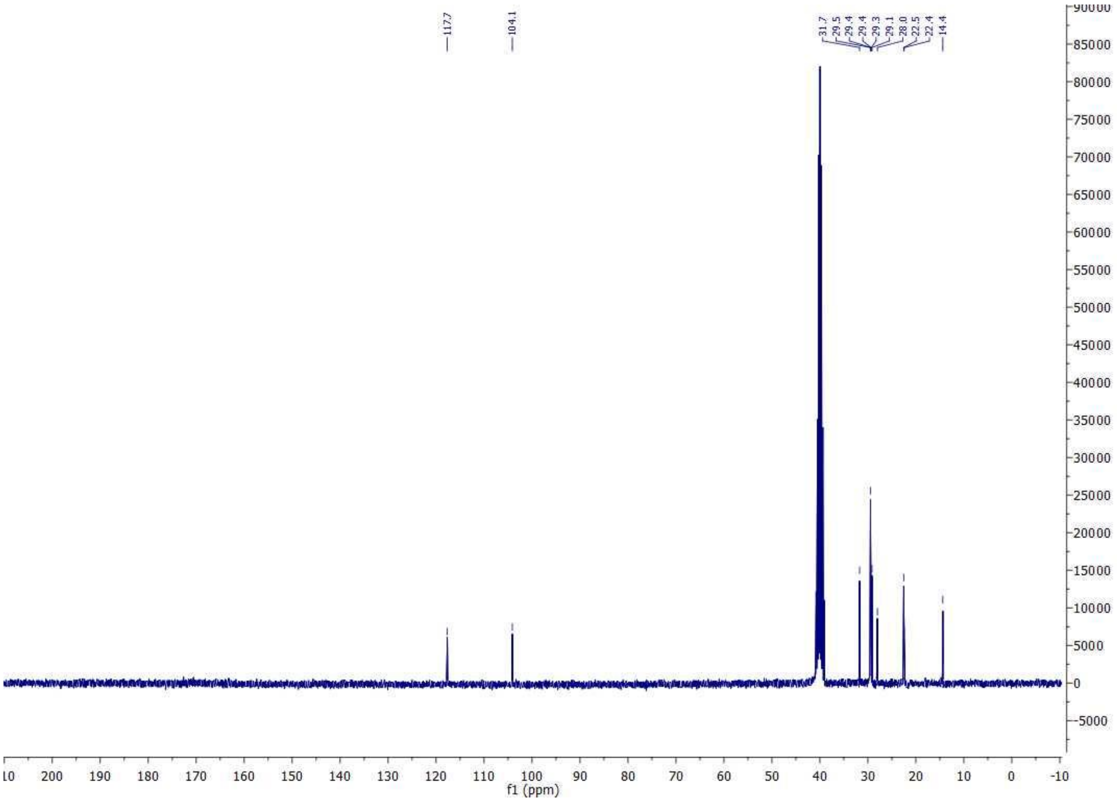


**Figure S8.** Rapanone (**2**) RMN 13C (300 Mz) in DMSO-d6.

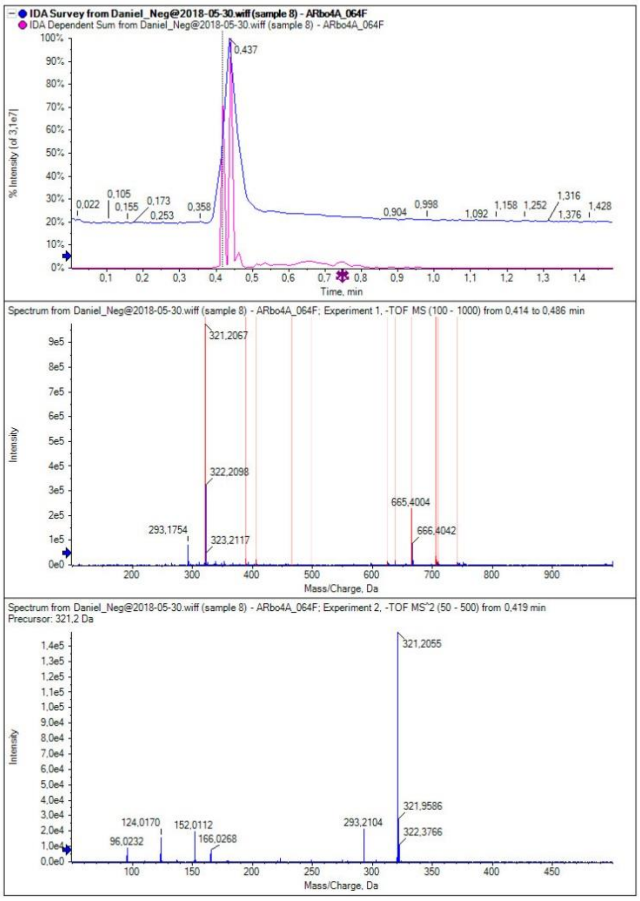


05/06/2018 17:55:14

**Figure S9.** Rapanone (**2**) MS.

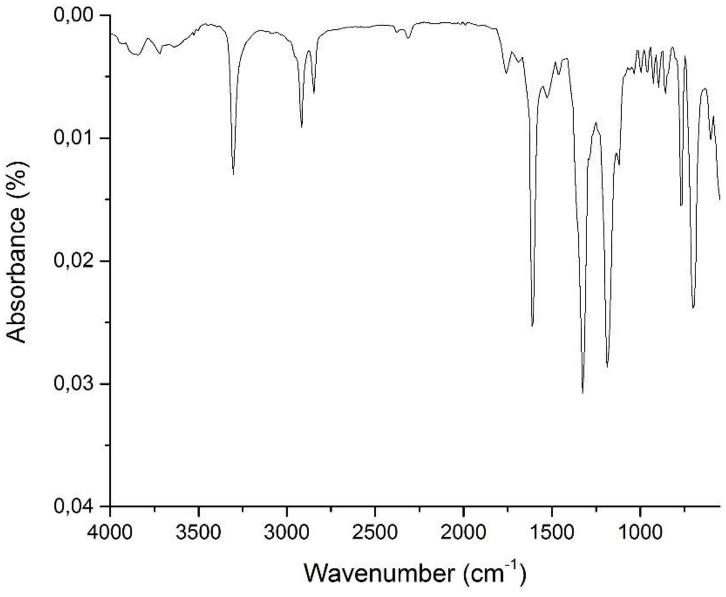


**Figure S10.** Rapanone (**2**) IR.

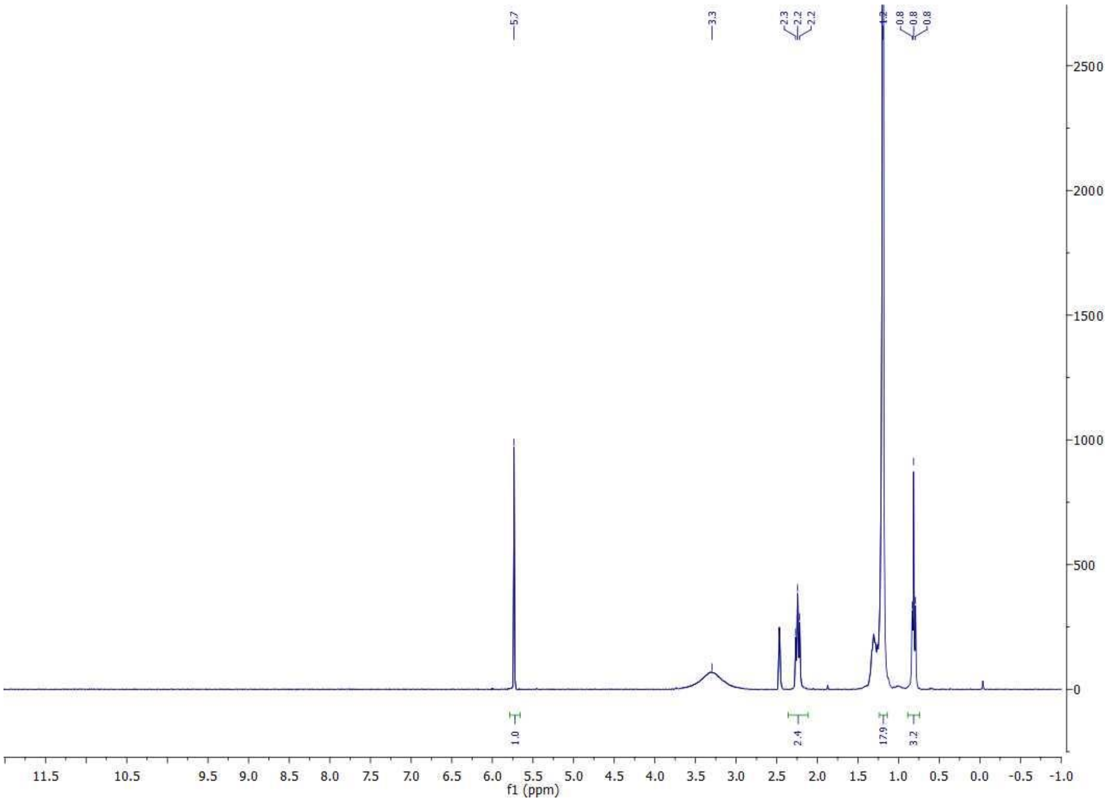


**Figure S11.** Embelin (**3**) RMN 1H (600 Mz) in DMSO-d6.

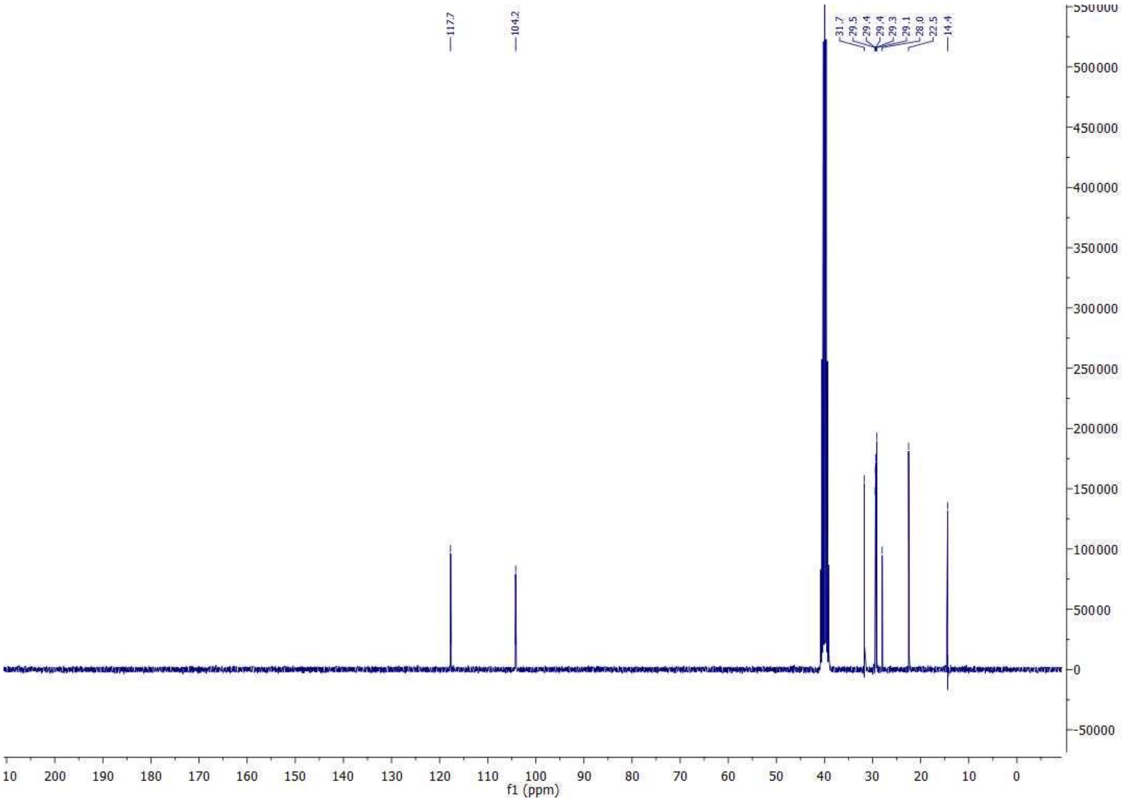


**Figure S12.** Embelin (**3**) RMN 13C (600 Mz) in DMSO-d6.

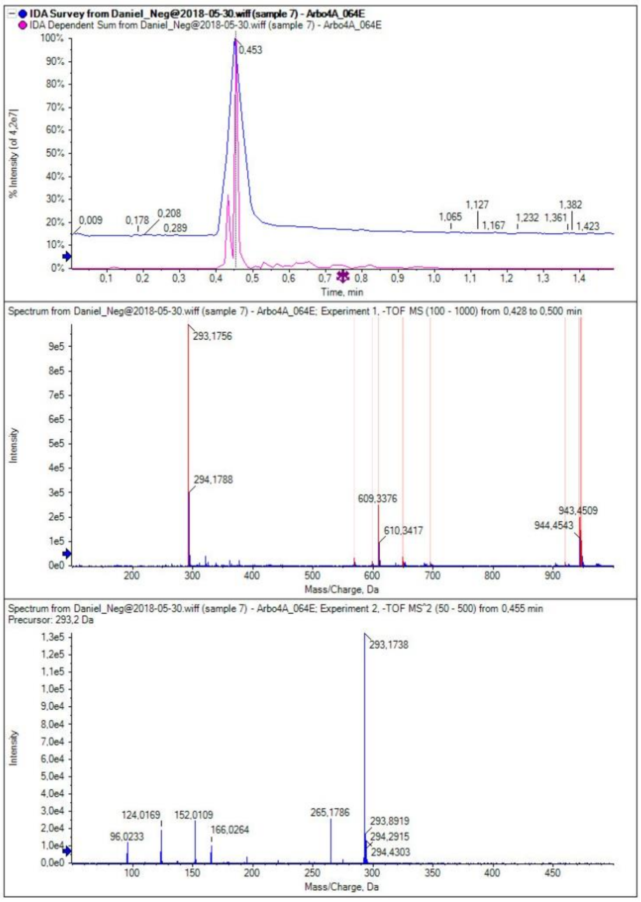


05/06/2018 17:47:26

**Figure S13.** Embelin (**3**) MS.

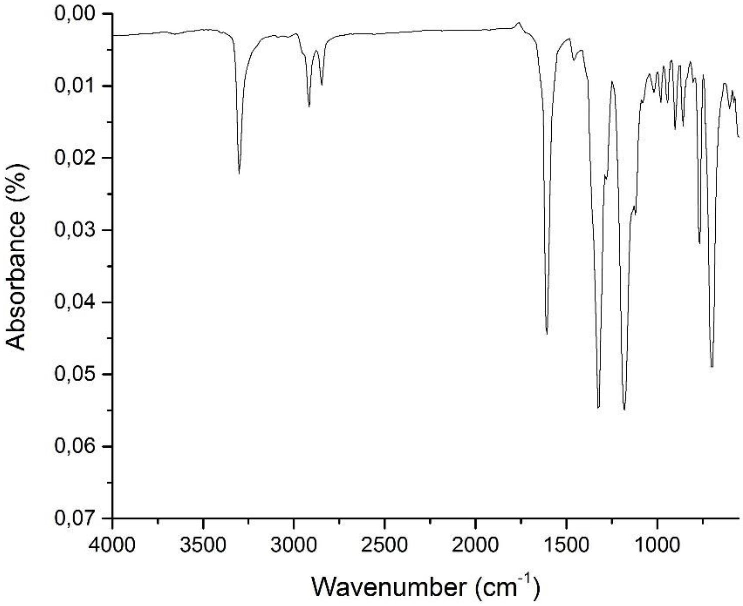


**Figure S14.** Embelin (**3**) IR.

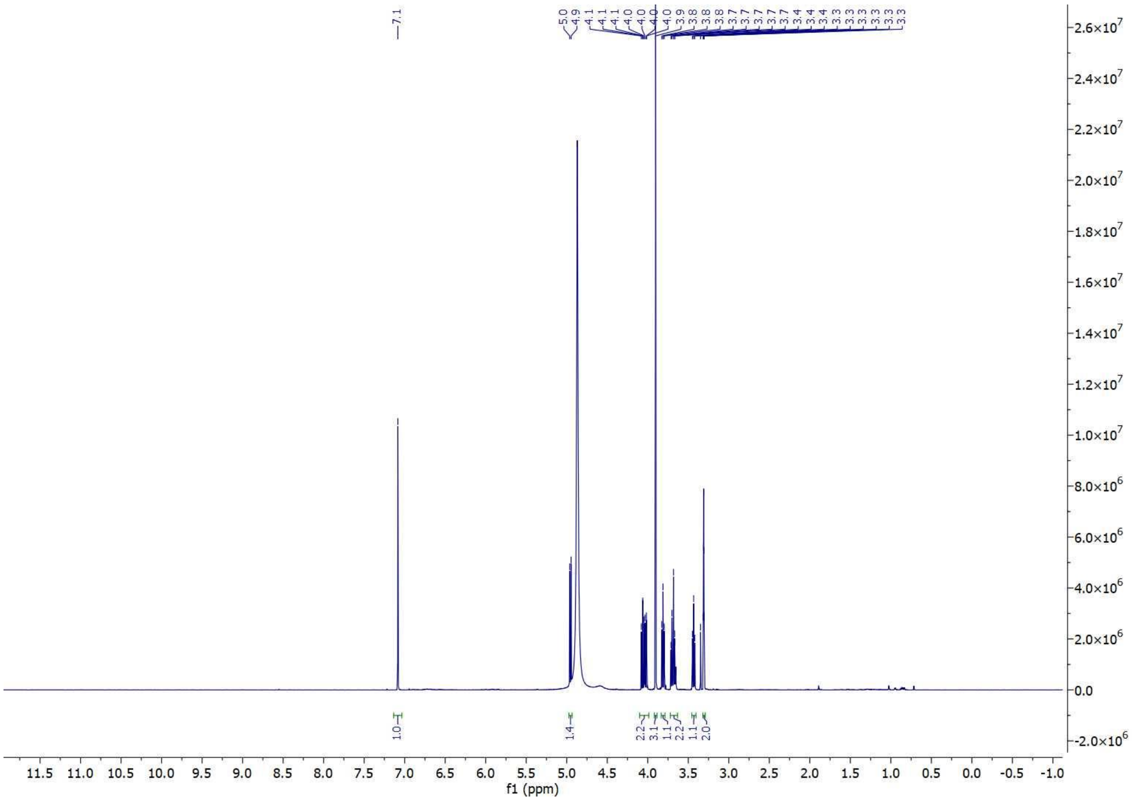


**Figure S15.** Bergenin (**32**) RMN 1H (600 Mz) in MeOD.

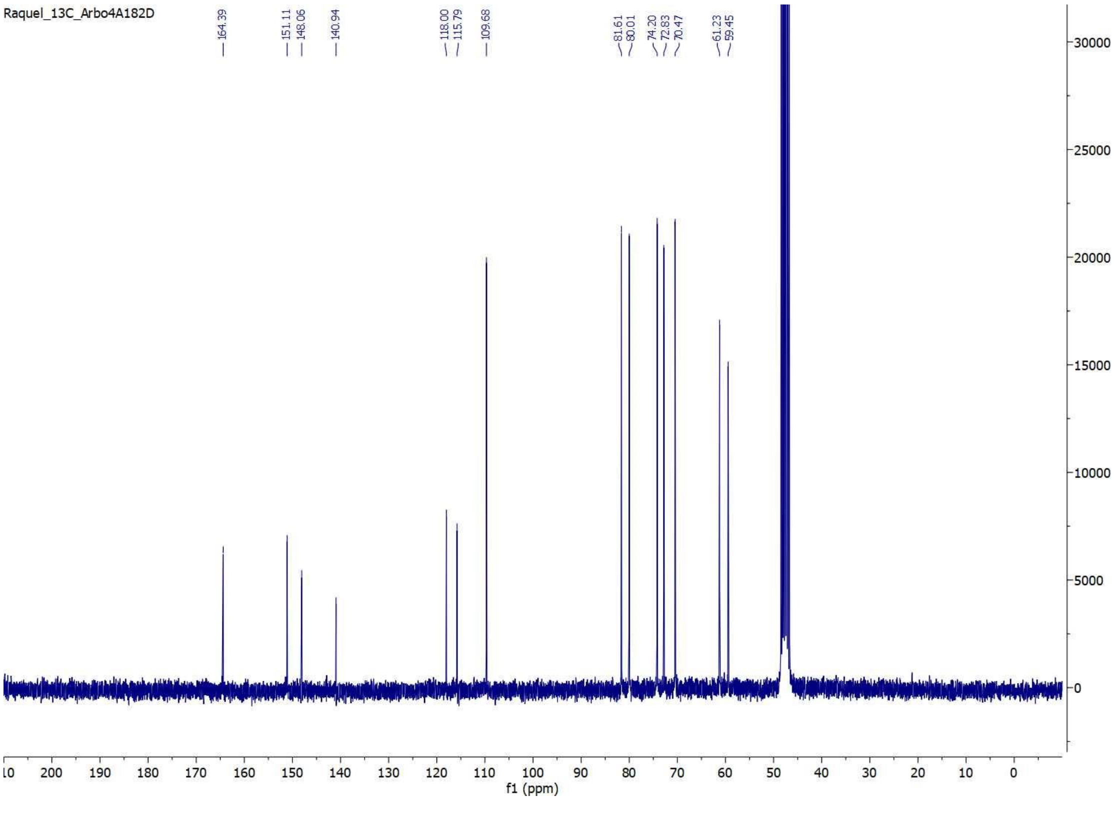


**Figure S16**. Bergenin (**32**) RMN 13C (300 Mz) in MeOD.

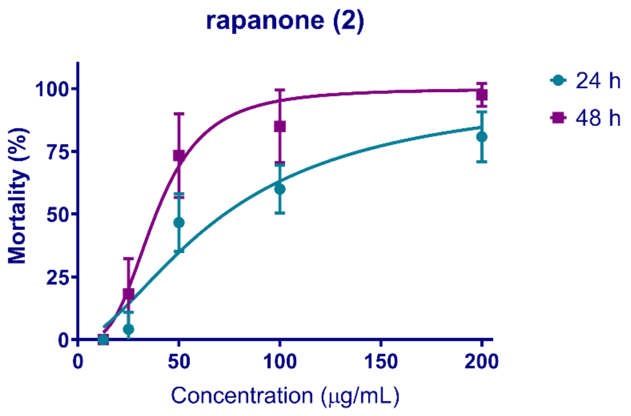


**Figure S17**. Rapanone (**2**) $LC_{50}$ (µg/mL). Final Volume = 3 mL. Number of larvae = 600.

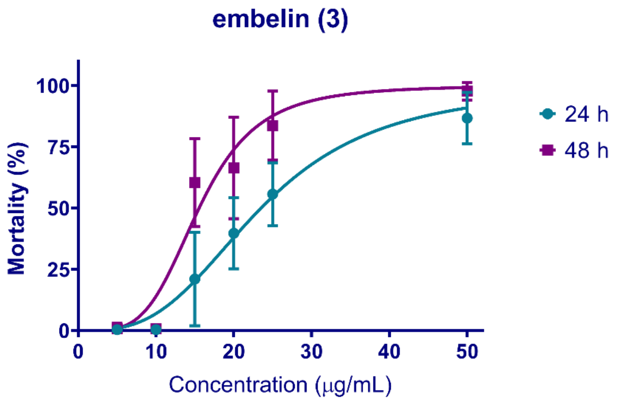


**Figure S18.** Embelin (**3**) $LC_{50}$ (µg/mL). Final Volume = 20 mL. Number of larvae = 1800.

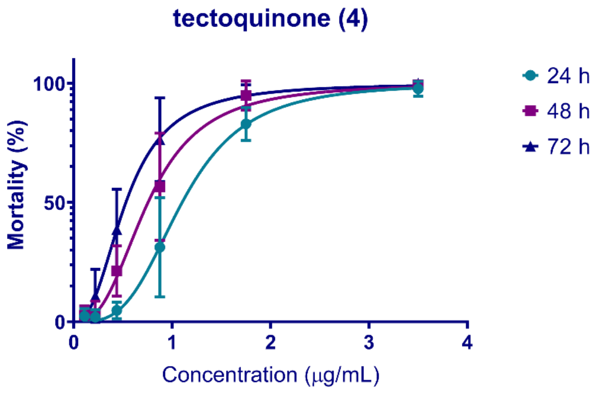


**Figure S19.** Tectoquinone (**4**) $LC_{50}$ (µg/mL). Final Volume = 120 mL. Number of larvae = 1800.

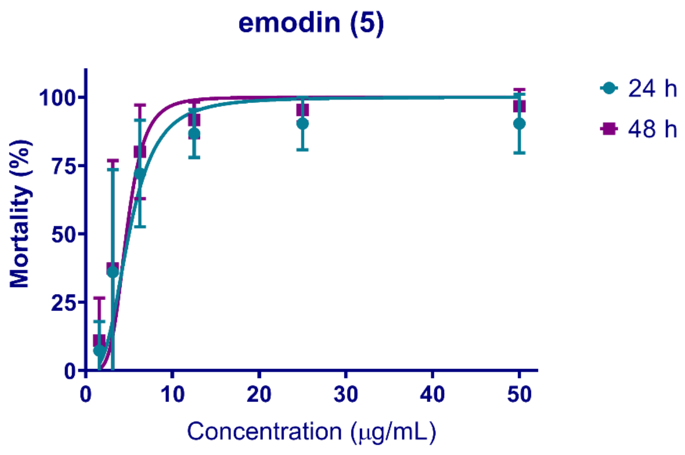


**Figure S20**. Emodin (**5**) $LC_{50}$ (μg/mL). Final Volume = 20 mL. Number of larvae = 1800.

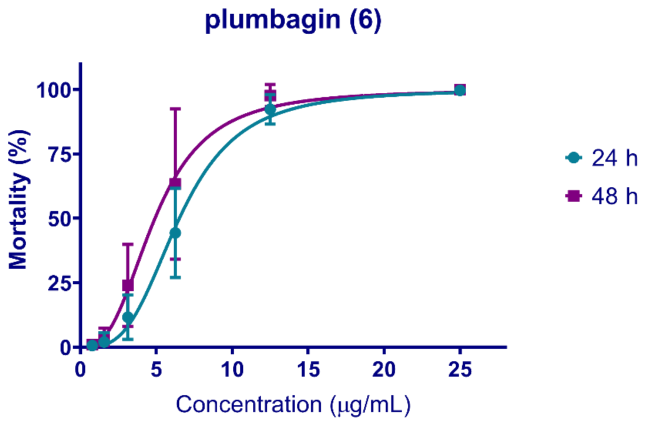


**Figure S21**. Plumbagin (**6**) $LC_{50}$ (μg/mL). Final Volume = 20 mL. Number of larvae = 1800.

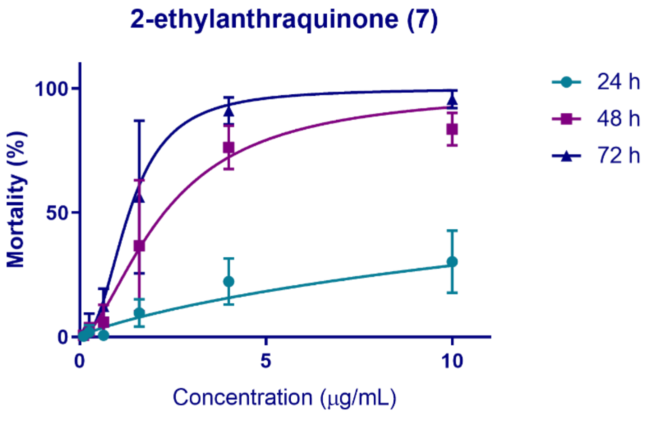


**Figure S22.** 2-ethylanthraquinone (**7**) $LC_{50}$ (μg/mL). Final Volume = 120 mL. Number of larvae = 1800.

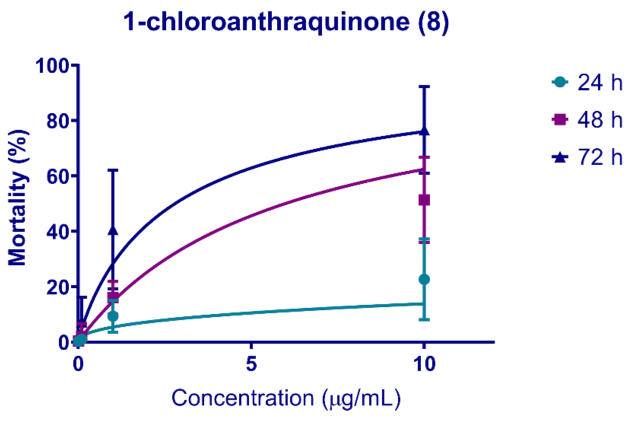


**Figure S23.** 1-chloroanthraquinone (**8**) $LC_{50}$ (µg/mL). Final Volume = 120 mL. Number of larvae = 1200.

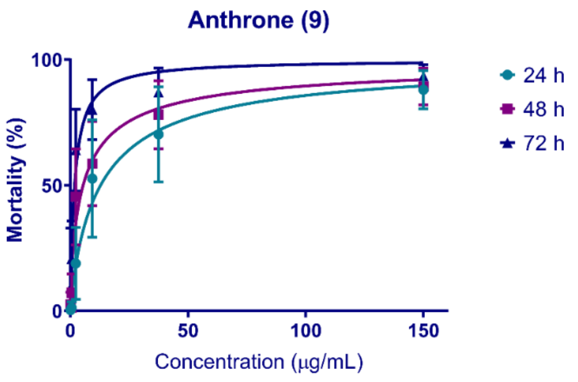


**Figure S24.** Anthrone (**9**) $LC_{50}$ (µg/mL). Final Volume = 120 mL. Number of larvae = 1800.

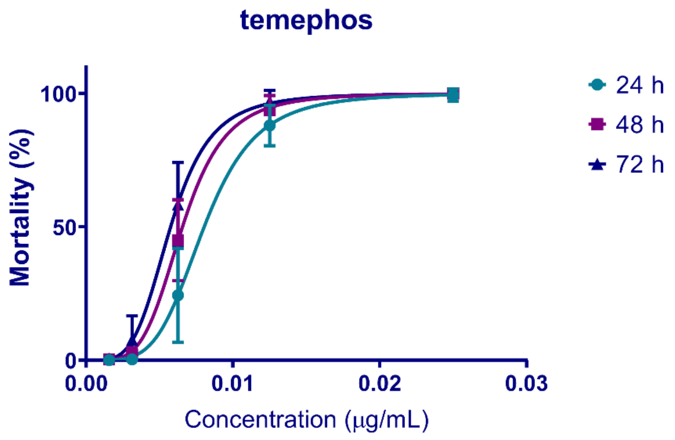


**Figure S25.** Temephos (positive control) $LC_{50}$ (µg/mL). Final Volume = 120 mL. Number of larvae = 1500.
